# Design and Optimisation of Bioactive Cyclic Peptides: Generation of a Down-Regulator of TNF Secretion

**DOI:** 10.3390/molecules191221529

**Published:** 2014-12-22

**Authors:** Roger New, Gurpal S. Bansal, Malgorzata Dryjska, Michal Bogus, Patricia Green, Marc Feldmann, Fionula Brennan

**Affiliations:** 1 Proxima Concepts Limited, c/o London Bioscience Innovation Centre, 2 Royal College Street, London NW1 0NH, UK; E-Mails: gurpal8@sky.com (G.S.B.); gosia@herbatabakwitnaca.pl (M.D.); michalbogus@proximaconcepts.com (M.B.); 2 Bone Medical Limited, 16 Ord Street, West Perth, WA 6005, Australia; 3 Kennedy Institute of Rheumatology, Roosevelt Drive, University of Oxford, Headington OX3 7FY, UK; E-Mails: patricia.green@kennedy.ox.ac.uk (P.G.); marc.feldmann@kennedy.ox.ac.uk (M.F.)

**Keywords:** cyclic peptide, TNF, interleukin 6, rheumatoid arthritis, lipo amino acid

## Abstract

Although strong binding interactions between protein receptor and ligand do not require the participation of a large number of amino acids in either site, short peptide chains are generally poor at recreating the types of protein-protein interactions which take place during cell recognition and signalling process, probably because their flexible backbones prevent the side chains from forming sufficiently rigid and stable epitopes, which can take part in binding with the desired strength and specificity. In a recently-reported study, it was shown that a proto-epitope containing F, R and S amino acids has the ability to down-regulate TNF secretion by macrophages. This paper extends these findings, putting those amino acids into a short cyclic peptide scaffold, and determining the optimal configuration required to overcome the problems of conformational instability, and give rise to molecules which have potential as therapeutic agents in human disease, such as rheumatoid arthritis.

## 1. Introduction

**Figure 1:**
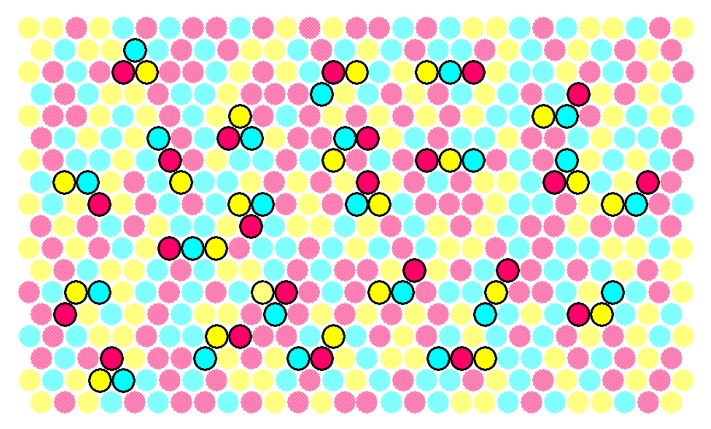
Representation of the distribution of amino acid headgroups on the surface of a micelle as described in reference [1], where the same combinations of amino acids can come together in many different configurations. One of these configurations will correspond to a structure with a desired biological activity.

As a result of conducting the Mozaic^TM^ discovery process (previously described [1]), it was discovered that when a combination of three amino acids (R, S & F) was presented to J774 cells (a murine macrophage cell line) on the surface of micelles, this preparation was highly effective in inhibiting secretion of TNF from these cells after they had been stimulated with cholera toxin B fragment (CTB) or lipopolysaccharide (LPS). The inhibition observed was specific to that particular combination of amino acids, other combinations usually having no effect, or in some cases having an opposite, stimulatory effect, even when the combination differed by only one amino acid (e.g., R, S & E [1]). It was concluded that the micelles were not inhibitory in their own right, but that the inhibition depended on interaction of the amino acids with a receptor on the cells. In particular, it is envisaged that the amino acids close-packed on the surface of the micelle can come together in many different configurations (see Figure 1), and it is reasoned that one of these configurations acts as a ligand, as it complements very closely the active site of a cell surface receptor, such that the binding of these two initiates a signal cascade which leads to a change in the behaviour of the cell.

It was thought most likely that the putative receptor was a protein expressed on the plasma membrane surface, since internalisation of the micelle would result in degradation processes separating the amino acids from each other, so specific interaction could not take place with receptors inside the cell.

It is well known that removal of TNF from the circulation using monoclonal antibodies can be a very effective method of treatment for rheumatoid arthritis [2], less well known outside industry is that TNF inhibitors are now the best-selling drug class. While reducing TNF secretion could also be a very effective therapeutic strategy not only in rheumatoid arthritis but in many other inflammatory diseases, the labile nature of micelles makes them poor candidates for a therapeutic agent, since they can readily be disassembled *in vivo*, for example by exchange of lipids with lipoproteins, adsorption onto large proteins, or fusion with cell membranes, followed by dispersion of the component amphiphiles. Furthermore, the amino acids on the micelle surface can adopt many different configurations, only one of which is likely to exert the desired biological effect *in vivo*. It is also possible that these additional configurations, while being inactive *in vitro*, may display other undesired side-effects *in vivo*, which could be counterproductive.

Thus, having used the Mozaic^TM^ technique to identify amino acids which can constitute a bio-active ligand when presented in combination, to use this information to generate a therapeutic entity, one must devise a molecular structure which is robust, unambiguous and free of moieties which may bind to receptors other than the intended target. In particular, the amino acid side chains must be presented to the putative receptor in exactly the same way as occurs when they are on the surface of the micelles. The experiments described here have been conducted in order to achieve this aim.

## 2. Results and Discussion

**Figure 2:**
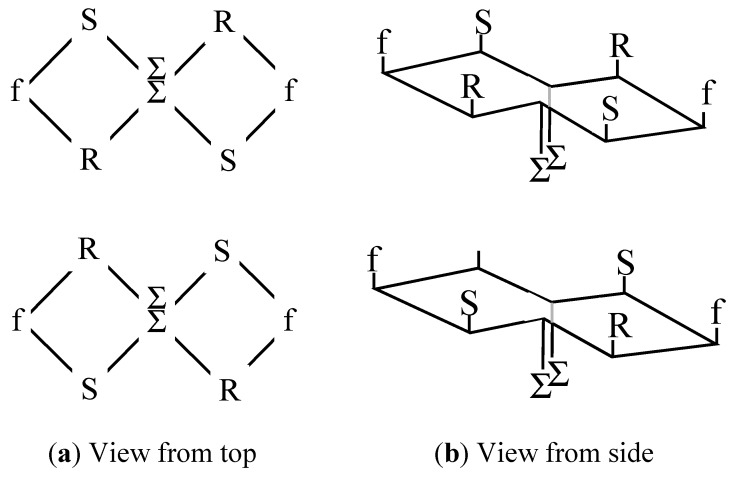
(**a**) General structure of the cyclic peptides employed in this paper, in which non-covalent interactions between the “Σ” amino acids bring them closer together; (**b**) View of the peptide ring from the side, showing showing the disposition of the side chains above and below the ring.

In order to mimic their disposition on the micelle surface, the amino acids need to be close together, and to be presented in a planar array. It was considered that the best way to achieve this was to link the peptides together in the form of a cyclic peptide. An additional condition which was thought may be important was to present the different amino acids in the form of a triangle, this being the simplest structure containing all three amino acids at the same time, all in direct contact with each other. Since a cyclic peptide of just three amino acids would be too constrained to form naturally, a hexapeptide was targeted instead. However, cyclic peptides without glycines or prolines tend to form very open rings, which would not give rise to any triangular structures. Consequently, an approach directed towards constraining the ring was employed, after its formation, in which a pair of extra amino acids was inserted on opposite sides of the ring. These amino acids had extended lipophilic side-chains, and could bring the two sides of the ring together by non-covalent association. The general structure of the resultant cyclic peptide is shown in Figure 2a, where the amino acid represented by “Σ” is an extended nor leucine, *i.e.*, H_2_N-CHR-CO_2_H, and R = (CH_2_)_9_-CH_3_.

Even-numbered cyclic peptide structures can be regarded as equivalent to small fragments of antiparallel beta-pleated sheets. In such sheets, the side chains are disposed alternately above and below the plane of the ring. In order to ensure that in this case all the functional side chains are located above the ring, and in the same plane, as on the surface of the micelle, with the lipophilic chains on the other side, face down, the chirality of the amino acids was adjusted so that that of the amino acids on the extreme left and extreme right of the ring was different from the remaining six (see Figure 2a). Thus, viewed from the side, the ring, with side chains, has the appearance as shown in Figure 2b. The scaffold of the type thus derived, comprising an internally constrained cyclic peptide, is described here as the Lexicon^TM^ backbone, and each of the peptides constructed using this backbone is designated with a “Lex” prefix.

**Figure 3:**
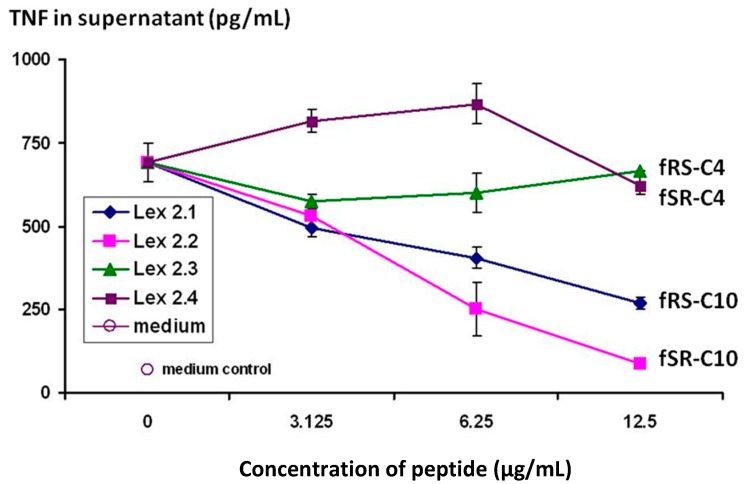
Changes in level of secretion of TNF into the supernatant of CTB-stimulated J774A.1 cells overnight after exposure to cyclic peptides as follows: Lex 2.1 and 2.2 are cyclo(-DF-R-Σ-S-DF-R-Σ-S-) and cyclo(-DF-S-Σ-R-DF-S-Σ-R-) respectively, where Σ represents an amino acid with a residue consisting of a straight C_10_ hydrocarbon chain. Lex 2.3 and 2.4 are cyclo(-DF-R-Nle-S-DF-R-Nle-S-) and cyclo(-DF-S-Nle-R-DF-S-Nle-R-) respectively, where norleucine (Nle) contributes a four-carbon sidechain.

The results shown in Figure 3 demonstrate that the cyclic peptides with the structure cyclo(-DF-R-Σ-S-DF-R-Σ-S-) and cyclo(-DF-S-Σ-R-DF-S-Σ-R-) (Lex 2.1 and 2.2 resp) do indeed have efficacy in terms of down-regulating the secretion of TNF from CTB-stimulated J774 cells, and that it displays this in a dose-dependent manner.

The importance of the “handedness” of the triangle is evident—*i.e.*, whether the sequence S-F-R in a clockwise or anticlockwise direction was best. As can be seen from the results, while activity was seen for both variants, R-F-S in a clockwise sequence (Lex 2.2) is more efficacious. The same figure also shows that, in order to exert this activity, lipophilic side chains of a certain length are essential, a C_4_ chain length as in norleucine (Lex 2.3 and 2.4) being insufficient.

**Figure 4:**
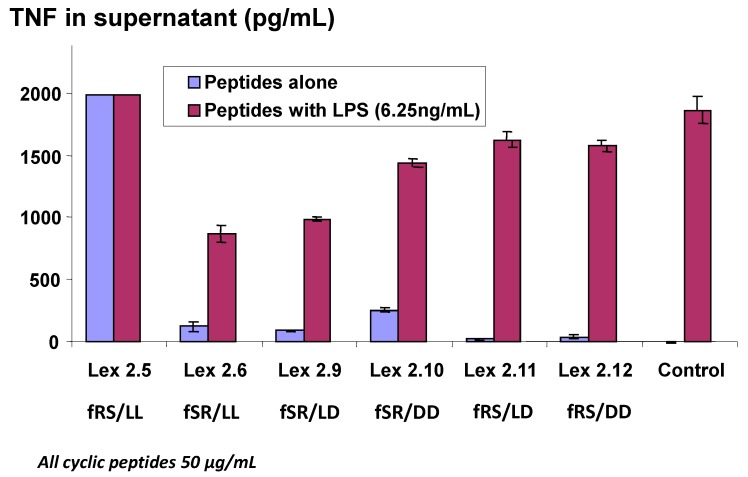
Changes in level of secretion of TNF into the supernatant of LPS-stimulated J774A.1 cells overnight after exposure to cyclic peptides as follows: Lex 2.5 and 2.6 are cyclo(dF-R-lC_10_-S-dF-R-lC_10_-S) and cyclo(dF-S-lC_10_-R-dF-S-lC_10_-R) respectively, Lex 2.9 and 2.10 are cyclo(dF-S-lC_10_-R-dF-S-dC_10_-R) and cyclo(dF-S-dC_10_-R-dF-S-dC_10_-R) respectively, and Lex 2.11 and 2.12 are cyclo(dF-R-lC_10_-S-dF-R-dC_10_-S) and cyclo(dF-R-dC_10_-S-dF-R-dC_10_-S), where C_10_ represents an amino acid with an extended C_10_ hydrocarbon chain.

In the previous experiment, although both the norleucines were in the “l” form, the alpha carbons of the long-chain lipo amino acids were racemic, being a random mixture of both “l” and “d” forms. Confirmation that best results are obtained when the two “Σ” lipo amino acids are in the l form is shown in the experiment reported in Figure 4, where all combinations of “Σ” l and d forms were tested in an experiment where very high levels of TNF were generated. It is worthy of note that Lex 2.5 consistently showed a strongly dose-related stimulatory activity in cells which otherwise appeared normal after exposure. This peptide, like Lex 2.6, has both lipo amino acids in the l form, but the order of amino acids in each domain, proceeding clockwise, is F-R-S, while in Lex 2.6 the order is F-S-R. For further experiments, Lex 2.6 was chosen for study, since this was seen to be the most efficacious structure tested, in terms of down-regulating TNF secretion.

**Figure 5:**
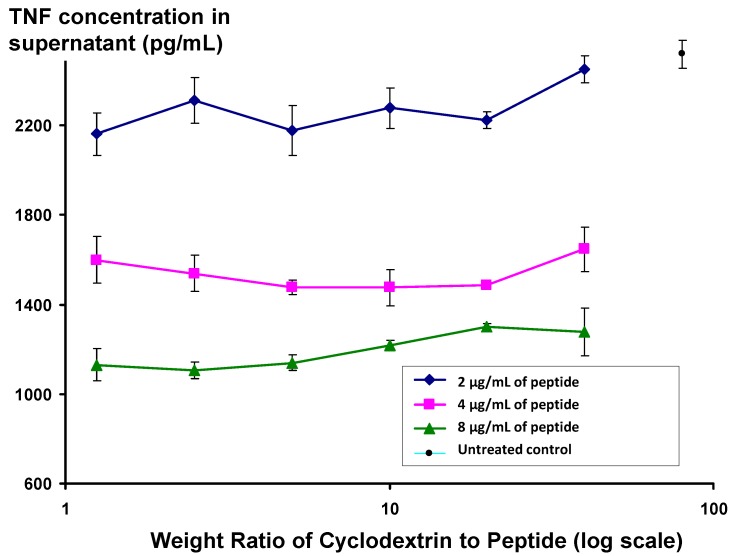
Changes in level of secretion of TNF into the supernatant of LPS-stimulated J774A.1 cells overnight after exposure to Lex 2.6 formulated with beta hydroxypropyl cyclodextrin at different ratios to peptide by weight.

Interestingly, bringing about association by linking the side chains together covalently, via a disulphide bridge, does not give the desired result (data not shown). In order to avoid problems with aggregation, which, it was feared, may affect the long-term stability of the product, a number of methodologies were employed to disperse the peptide prior to introduction into aqueous medium. Dissolution in HFIP, followed by drying down and addition of water worked reasonably well, as did dissolving in transcutol at high concentration, then adding to water. The most effective approach, however, was to dissolve the peptide in either of these solvents in the presence of β-hydroxypropyl cyclodextrin. Detailed methodology is given in the Experimental section. At all levels of cyclodextrin employed, increased homogeneity was achieved, as evidenced by a significant reduction in light scattering of the preparation. Interestingly, as is seen in Figure 5, the activity of peptide appears to be independent of the ratio of peptide to cyclodextrin, even at low cyclodextrin concentrations, where the cyclodextrin:peptide weight ratio is 1.25, equivalent to a molar ratio of 1.3.

The ability of Lex 2.6 to inhibit secretion of TNF from CTB-stimulated J774 cells is an encouraging sign that it may also have an effect on TNF production in rheumatoid arthritis in humans, where TNF is a major contributing factor in perpetuating the inflammatory condition. However, J774 cells are murine-derived macrophages, and since the down-regulation is probably a receptor-mediated process, it is necessary to confirm that Lex 2.6 acts on the human receptor in the same way as it does on murine receptors. Of greater concern is the fact that the agents used to stimulate TNF secretion (CTB and LPS) are very different from those which stimulate macrophages in the arthritic joint. In order to test our peptide under circumstances more reminiscent of the human clinical situation, the *in vitro* model of Cytokine activated T cells are co-cultured with monocytes, developed by Brennan *et al.* [3] was employed. Human T cells are incubated in a cytokine cocktail similar to that pertaining in an arthritic joint, generating a T-cell phenotype which stimulates human macrophages to secrete TNF [4,5]. The details of this technique are described in the Materials and Methods section.

**Figure 6:**
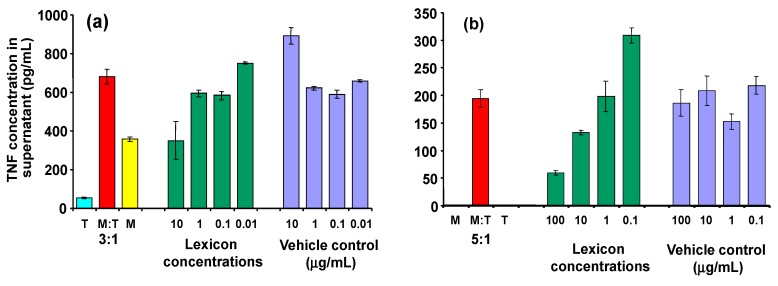
Effect of Lex2.6 at different concentrations on TNF secretion of cells in the cognate assay. (**a**) Lex 2.6 formulated with cyclodextrin in the presence of transcutol; (**b**) Lex 2.6 formulated with cyclodextrin in absence of transcutol.

As can be seen in Figure 6, inhibitory activity is seen clearly at a dose of 10 µg/mL (panel a). At high concentrations, the control (transcutol/CD alone) also affected the macrophages, so interpration of the results at higher doses is difficult. Similar effects were observed when transcutol was omitted at the processing stage (panel b), although the CD alone still seems to exert an effect on the macrophages in its own right.

**Figure 7:**
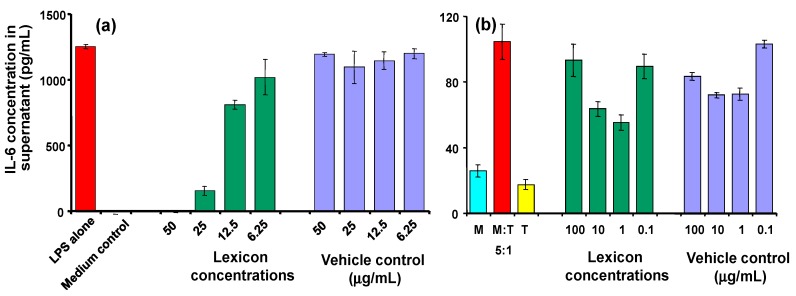
(**a**) Effect of Lex 2.6 at different concentrations on IL-6 secretion of LPS-stimulated J774A.1 cells; (**b**) Effect of Lex2.6 at different concentrations on IL-6 secretion of cells in the cognate assay. In these experiments, Lex 2.6 was formulated with cyclodextrin in the absence of transcutol.

In addition to TNF, the effect of Lex 2.6 on other cytokines was investigated (Figure 7). Clear dose-related inhibition of IL-6 secretion is seen with LPS-stimulated J774A.1 cells (panel 7(a)), and similar trends are observed in the cognate model (panel 7(b)), where reductions in IL-6 secretion at 10 and 1 μg/mL peptide were seen, relative to the corresponding vehicle controls. An MTT test was carried out on replicates of each group, the results of which confirmed that there was no reduction in cell metabolic activity, indicating that the reduction in cytokines seen were not due to cell death or general reduction in viability. No effect was seen on levels of IL-1 or IL-10 in the cognate model.

### Discussion

Linear peptides can often interact well with protein binding sites, and because of their flexibility, can readily adapt to a structure on a receptor molecule to achieve a good fit. The same flexibility, however, means that such peptides may lack specificity, being able to adapt to varied binding sites in many different molecules. It is also difficult to envisage how such flexible structures may bind strongly with other structures of similar flexibility—for example to other linear peptides, or to peptides loops. In such cases, introduction of conformation rigidity will be advantageous, and this can often, also, increase the strength of the desired binding interaction. In a series of studies conducted in the labs of Martin and coworkers [6,7], it is shown that in general a reduction in conformational flexibility is associated with an increase in Gibbs free energy of binding to ligands, although, surprisingly, it is often the change in enthalpy which contributes most to this effect, rather than entropy, as was originally assumed. A standard approach directed towards strengthening binding interactions in this way is to employ peptides in a cyclic form, since the conformational flexibility of the peptide backbone is reduced. The success of this strategy has been demonstrated by Udugamasooriya and Spaller [8], as well as by DeLorbe *et al.* [9], where ligand binding interactions of a series of peptides in both linear and cyclic forms have been compared. The advantages and limitations of linear *versus* cyclic peptides are reviewed by Roxin and Zheng [10].

In simple peptides, cyclisation introduces a geometrical constraint, resulting, under normal circumstances, in a buckled ring conformation; there is still a high degree of flexibility, however, and multiple configurations can form, where different pairs of amino acids approach closely at the buckle position (five configurations in a decapeptide for example). Perhaps for these, and other reasons, few cyclic peptides have successfully been employed as therapeutic agents modifying cell behaviour. One exception is valinomycin, but in this case, the sequence contains two prolines, which encourage the formation of beta turns, thus fixing the ring in one single conformation. Another example is cyclosporin, where the presence of glycine, in conjunction with N-methylation of peptide nitrogens similarly induces formation of beta turns. Two interesting new classes of cyclic peptides have recently been described [11]—cyclotides and conotoxins; in both cases these are stabilised by the presence of multiple disulphide bridges, and the linear and cyclised versions of the peptide chain in general have identical tertiary structure. Thus, in these peptides, cyclisation itself does not induce a geometrical constraint.

To avoid the possibility of multiple conformations being present, it is necessary to engineer an association across the ring in which one pair of amino acids locates itself preferentially at the buckle position, pulling the sides of the ring closer together. In the system described here, this has been achieved by introducing lipo-amino acids on opposite sides of the ring, whose side-chains can interact via non-covalent association.

An additional measure employed here to increase the rigidity still more is to complex the peptide with cyclodextrin. This will help to keep the hydrocarbon chains close together, as they are drawn into the cyclodextrin cavity, perhaps increasing their proximity yet further, and at the same time causing the buckled ring to flatten out, adopting a more planar structure capable of mimicking the surface of the micelle, which provided the first signs of biological activity for the amino acid combination identified.

The inflammatory action in the arthritic joint is thought to be the result of a vicious circle of mutual stimulation by T-cells and macrophages [12,13,14]. In this scenario, secretion of TNF and other cytokines by synovial macrophages encourages the persistance of an inflammatory phenotype, which in turn stimulates macrophages to continue secretion of pro-inflammatory cytokines. The key role that TNF plays in this scenario is highlighted by the fact that reduction of TNF levels alone, in the bloodstream and other body fluids, using anti-TNF monoclonal antibodies, is enough to reduce dramatically the symptoms of rheumatoid arthritis in the clinic [15].

In the approach taken here, rather than try to remove TNF from the tissues after it has been secreted, the aim is to act on cells in order to prevent its being secreted in the first place. Evidence to be presented separately suggests that Lex 2.6 inhibits secretion from macrophages alone, and has no effect on secretion by lymphocytes. Retention of the ability of lympohcytes to secrete TNF could be important in avoiding some of the side-effects seen with standard TNF blockage. Two conditions known to be associated with anti-TNF antibody treatment of rheumatoid arthritis, namely increased infection with *M. tuberculosis* and occurrence of Merkel cell carcinomas, have both been identified as being kept at bay by TNF-secreting lymphocytes [16,17] respectively.

The fact that Lex 2.6 inhibits secretion of TNF when the latter is elicited by a variety of methods (CTB, LPS, modified T-cell phenotype) suggests that it is working at a profound level on macrophage activation in general, rather than just a single TNF-related pathway. Separate experiments (data not shown) have demonstrated that, in human buffy-coat macrophages, reduction in TNF secretion by Lex 2.6 precursors is accompanied by a reduction in cytosolic TNF-specific mRNA, indicating that inhibition of TNF secretion is the result of halting TNF production inside the cell, rather than simply a block in translocation across the plasma membrane, from cytosol to the exterior.

In the studies conducted here, an IC_50_ of about 4uM was observed for the Lex2.6/cyclodextrin complex, and the IC_10_ is probably in the high nanomolar range. However, in these experiments the stimulus was, of necessity a very strong one, in order to achieve a reproducible level of secretion, and it is possible that *in vivo*, where the stimulation, although chronic, is of a milder nature, the impact of the molecule on the cells would be greater at lower concentrations. *In vivo* studies in animal models for RA, to be reported elsewhere, suggest that this is indeed the case, and give encouragement that Lex2.6 could be effective in treatment at a commercially viable dose level.

The ability of Lex 2.6 to affect the secretion not only of TNF, but of IL-6 also, is perhaps not surprising since these pathways are often interlinked, and it is possible that one causes the secretion of the other. However, the fact that both of these can be reduced by a single agent - Lex 2.6—is another encouraging factor suggesting efficacy of this peptide in the human disease, since it has been shown that interfering with the activity of either one of these cytokines alone has an effect on the disease, in the case of IL6, reducing the destructive effects on synovial fibroblasts. [18,19,20]. The results of experiments to identify the receptor responsible for down-regulation of secretion of these cytokines will be reported elsewhere.

## 3. Experimental Section

### 3.1. Reagents

Peptides: Cyclic peptides were synthesised by Peptide & Protein Research Ltd, Fareham, UK using standard methods (solid phase Fmoc chemistry, followed by cyclisation in solution). This company also synthesised the racemic and chiral lipo-amino acids employed. Transcutol (diethylene diglycol), hexafluoro isopropanol (HFIP) and β-hydroxypropylcyclodextrin (CD) were all purchased from Sigma UK Ltd. (Gillingham, UK).

J774A.1 Cell culture: Dulbecco’s Minimal Essential Medium (DMEM) and foetal bovine serum (FBS) were purchased from Invitrogen (Paisley, UK). Pen/Strep solution (25 units/mL) and l-glutamine (20 mM) were obtained from Gibco UK (Paisley, UK). Cholera Toxin B fragment (CTB) and *E. coli* lipopolysaccharide (LPS) were purchased from Sigma UK Ltd. The ELISA kit for detection of Tumour Necrosis Factor (TNF) was purchased from R&D Systems Ltd (Abingdon, UK). Cells of the J774A.1 line were purchased from the European Cell Culture Collection at passage number 14.

### 3.2. Cell Culture (J774A.1 Macrophage Cell Line)

Cells between 40 and 60 passage number were maintained in culture at 37 °C in DMEM supplemented with 10% Foetal Bovine Serum (FBS), 1% Pen/Strep solution and 1% glutamine in a 5% CO2/air atmosphere. For each experiment, cells were scraped gently from the surface of the culture vessel and re suspended in medium without FBS, at a concentration of 0.2 × 10^6^ cells/mL. The cell suspension was then transferred to each well of 24-well cluster plates (1 mL per well). The cells were incubated overnight to give a sub-confluent, adherent monolayer, whereupon the medium was replaced with fresh medium containing micelles at the desired concentrations. The cells were, incubated overnight and the TNF concentration in the supernatant was measured the following day using a sandwich ELISA kit. In some experiments, immunostimulants (CTB, 10 µg/mL or LPS between 0.0125 and 0.1 µg/mL final concentration) were added to the wells prior to overnight incubation, four hours after administration of the micelles.

### 3.3. Cytokine-Activated T-Cells (Tck)

A methodology described by Brennan *et al.* [3] was employed. Peripheral blood lymphocytes (>95%) are enriched by Centrifugal Elutriation from plateletphoresis blood cones. CD45RO^+^ Tcells (>98% purity) isolated by negative selection (Miltenyi, Bisley, UK) are re-suspended to a density of 1 × 10^6^ cells/mL in RPMI 1640 containing 10% heat-inactivated AB^+^ Human Serum. Cytokines are added to the cells at the following concentrations, hIL-2 @25 ng/mL, hIL-6 @100 ng/mL and hTNFα @25 ng/mL, all purchased from Peprotech UK (London, UK). The cells are incubated at 37 °C, 5% CO_2_ for 8 d after which they are pelleted twice at 1500 rpm for 5 min, counted and then used in a cognate experiment.

### 3.4. T Cell/Monocyte co-Culture (Cognate)

A methodology described by Brennan *et al.* [3] is employed as follows. Elutriation-enriched (>95%) autologous monocytes are cultured at 1 × 10^6^ cells/mL (100 microlitres) in RPMI 1640 containing 5% heat-inactivated FCS, in a 96-well, round-bottomed culture plate. After adjusting the cell number, cytokine activated T-cells (Tck) are added to the monocytes at a 5:1 ratio, the peptides are then added into the appropriate wells, with a final volume of 200 μL/well. Cells are co-cultured at 37 °C, 5% CO_2_ for 18 h after which the supernatants are removed and assayed for cytokine production by ELISA.

### 3.5. MTT Viability Assay

After removal of the supernatants 100 μL of RPMI 1640 is added to each well of the plate. 10 μL of MTT dye (stock 5 mg/mL) is added to each well and incubated for 4 h at 37 °C 5% CO_2_, the MTT crystals are dissolved with SDS after which the plates are read at 620 nm.

## 4. Conclusions

This paper, and the earlier publication in the series [1], together describe a new approach to identifying and creating bioactive molecules which can have application in any therapeutic, diagnostic or other situation where a putative binding interaction takes place. The first stage of the method involves presenting building blocks on the surface of micelles, in order to determine which combination of building blocks may elicit a biological response. However, in order for this information to be translated into a viable structure which can function in a biological environment, a scaffold needs to be designed capable of presenting the building blocks in a biologically active form. The cyclic peptides described here constitute such a scaffold, and we have shown the importance of inducing constraints in the ring in order to achieve maximal bioefficacy. It is hoped that the findings presented here will serve as a generic method for creating, rapidly and cost-effectively, small stable bioactive peptides with application in a wide range of medical and other scenarios.
